# Highlight selection of radiochemistry and radiopharmacy developments by editorial board (January–June 2020)

**DOI:** 10.1186/s41181-020-00118-5

**Published:** 2021-01-28

**Authors:** Mohammed Al-Qahtani, Martin Behe, Guy Bormans, Giuseppe Carlucci, Jean Dasilva, Clemens Decristoforo, Philip H. Elsinga, Klaus Kopka, Xiang-Guo Li, Robert Mach, Oskar Middel, Jan Passchier, Marianne Patt, Ivan Penuelas, Ana Rey, Peter J. H. Scott, Sergio Todde, Jun Toyohara, Danielle Vugts

**Affiliations:** 1 King Faisal Specialist Hospital and Research Center, Riyadh, Saudi Arabia; 2 Paul Scherrer Institute, Villigen, Switzerland; 3 Katholieke Universiteit Leuven, Leuven, Belgium; 4 UCLA Molecular and Medical Pharmacology Department, Los Angeles, USA; 5 University of Montreal, Montreal, Canada; 6 Universitaetsklinikum fur Nuclearmedizin, Innsbruck, Austria; 7 Nuclear Medicine and Molecular Imaging, University Medical Center Groningen, University of Groningen, Hanzeplein 1, 9713GZ Groningen, The Netherlands; 8 Helmholtz Zentrum Dresden Rossendorf, Dresden, Germany; 9 Turku PET-center, Turku, Finland; 10 University of Pennsylvania, Philadelphia, USA; 11 St Olavs Hospital and Norges teknisk-naturvitenskapelige universitet (NTNU), Trondheim, Norway; 12 Invicro, London, UK; 13 University of Leipzig, Leipzig, Germany; 14 University Clinic of Navarra, Pamplona, Spain; 15 Universidad de la Republica, Montevideo, Uruguay; 16 University of Michigan, Ann Arbor, USA; 17 Tecnomed Foundation, University of Milano - Bicocca, Milan, Italy; 18 Tokyo Metropolitan Institute of Gerontology, Tokyo, Japan; 19 Amsterdam UMC, Amsterdam, The Netherlands

## Abstract

**Background:**

The Editorial Board of EJNMMI Radiopharmacy and Chemistry releases a biyearly highlight commentary to describe trends in the field.

**Results:**

This commentary of highlights has resulted in 19 different topics selected by each member of the Editorial Board addressing a variety of aspects ranging from novel radiochemistry to first in man application of novel radiopharmaceuticals.

**Conclusion:**

Trends in radiochemistry and radiopharmacy are highlighted demonstrating the progress in the research field being the scope of EJNMMI Radiopharmacy and Chemistry.

## Introduction

As a new initiative each individual member of the Editorial Board has selected a highlight article that has appeared in the radiochemistry and radiopharmacy literature during the period January–June 2020. The aim of this collaborative initiative is to create a biyearly overview summarizing the latest trends in the field.

^**18**^**F-Labelled folate tracer now in the clinic, finally**

*By Xiang-Guo Li*

Folic acid is a vitamin and it has high affinity to folate receptors which are upregulated in inflammatory diseases as well as cancers. It is well justified to utilize folate-based ligands for targeted imaging with positron emission tomography (PET) techniques, and many types of radiolabeling strategies have been proposed by the research community during the long journey of radioligand development. Recently, a folate tracer named as [^18^F]fluoro-PEG-folate has been evaluated in patients with rheumatoid arthritis (Verweij et al. 2020). This radiopharmaceutical has been prepared via a classic prosthetic compound N-succinimidyl-4-[^18^F]fluorobenzoate. [^18^F]Fluoro-PEG-folate proved safe for human use and feasible for PET imaging rheumatoid arthritis by targeting folate receptors on macrophages.

**Copper-64 chloride is an attractive clinical tool**

*By Oskar Middel*

This uncomplicated and well-written article (Kjærgaard et al. 2020), suitable for educational purposes, describes the biodistribution, dosimetry and kinetics of the ^64^Cu^2+^ ion in healthy volunteers. The researchers used their opportunities to demonstrate and visualize the differences in kinetics and biodistribution in oral and intravenous administrations. This study shows negligible excretion of ^64^Cu, and the appropriate organ absorbed doses and effective dose. As a benchmark for other research and follow-up studies, the introduction is a good and complete overview additionally describing the bio-pathway of the ^64^Cu^2+^ ion. Therefore, the article serves as good reference and overview for both clinical research using ^64^CuCl_2_, as for research using the theranostic approach.

**Droplet radiochemistry**: **next generation technology**

*By Giuseppe Carlucci*

The expensive equipment and resources required for the production of PET imaging tracers make it challenging to economically produce batches for research studies in the pre-clinical phase. Progress in miniaturization and automation of radiosynthesizers was described based on droplet microfluidics (Lisova et al. 2020), demonstrating that O-(2-[^18^F]fluoroethyl)-L-tyrosine ([^18^F]FET) can be produced in just over half the time and with 100-fold less reagents and isotope compared to conventional methods, while providing a probe with high molar activity and in sufficient quantity to support complex, multi-animal dynamic imaging studies. Other recent papers by this group (Lisova et al. 2018) show that this approach can be applied to synthesize different tracers, and that the quantity can be scaled up to produce clinical doses, suggesting the possibility for droplet radiochemistry to serve as a more convenient and resource-efficient way to synthesize PET tracers.

**Next important step in pretargeting strategy**.

*By Philip Elsinga*

Tumor-targeting imaging agents with slow pharmacokinetics often display low tumor uptake. To circumvent this problem, a pretargeting strategy was used applying Peptobrush-TransCycloOctene (TCO) targeting the tumor making use of the EPR effect followed by administration of an ^111^In-labelled tetrazine to react in vivo through an Inversed Electron-Demand Diels-Alder reaction (IEDDA). TCO groups suffer from in vivo degradation resulting in reduced IEDDA-reactivity. The innovative aspect of this work (Steen et al. 2020) is the creation of PeptoBrush-TCO polymers that prevent TCO from degradation as they have a hydrophilic shell and stabilizing TCO-containing hydrophobic patches still accessible for IEDDA. Under optimal conditions, 77-fold enhancement of IEDDA-reactivity was found compared to small-molecule TCOs. Sufficient tumor-to-background ratios were obtained starting from 2 h after injection of the radiolabeled tetrazine.

**Elegant incorporation of a dye structure in the molecular design of a hybrid imaging tracer**

*By Clemens Decristoforo*

Hybrid tracers combining Near Infrared (NIR) imaging with SPECT or PET have gained increasing interest over the last few years. A major challenge is the incorporation of large NIR dyes having detrimental impact on pharmacokinetics and target interaction. A series of PSMA targeting, ^99m^Tc-labelled compounds was designed using different cyanine dyes as linkers between a EuK PSMA targeting sequence and a MAS_3_-Technetium chelator (Hensbergen et al. 2020). The authors took advantage of the lipophilicity of the dye using it as pharmacophore. The approach resulted in analogues with good tumour identification by both SPECT and fluorescence imaging. This elegant example shows the importance of a clever molecular design to modify and optimize target interaction and pharmacokinetics particularly in the development of hybrid imaging agents.

**Combination therapy – the way to go with targeted radiopeptide therapy (TRT)**

*By Martin Behe*

This paper (Cullinane et al. 2020) describes the enhancement of the tumor treatment efficacy by the combination of a targeted radiopeptide therapy (^177^Lu[Lu]DOTATATE) with an inhibitor of the enzyme Poly-ADP-Ribose-Polymerase (PARP). This is a follow up study with a more potent PARP inhibitor Talazoparib from a paper published by the Rotterdam group (Nonnekens et al. 2016). PARP is involved in repairing single strain DNA breaks as they are induced mainly with β^−^-emitters. They first evaluated different endocrine cell lines for the somatostatin receptor 2 (sstr2) expression in vitro as well as for the in vivo uptake with ^68^Ga[Ga]DOTATATE. They tested the combination therapy of Talazoparib and ^177^Lu[Lu]DOTATATE in mice bearing AR42J s.c. tumors. Significant slower tumor growth and longer survival compared to ^177^Lu[Lu]DOTATATE as a monotherapy was observed. The monotherapy with the PARP inhibitor alone showed no differences compared to the control group.

This paper shows that there is still big potential to improve efficacy of targeted radionuclide therapy with “intelligent” combinatory approaches. The paper nicely examined different aspects of the radionuclide therapy, but it would benefit from a more detailed evaluation of the biological aspects of PARP inhibition (e.g. PARP expression or examine downstream pathway changes). In order to realize the full potential of combination therapy, it is extremely important that we better understand and study the biological aspects of the therapeutic process.

**An efficient and simplified method for nucleophilic aromatic and aliphatic** ^**18**^**F-fluorination**

*By Jun Toyohara*

**Fig. 1:**
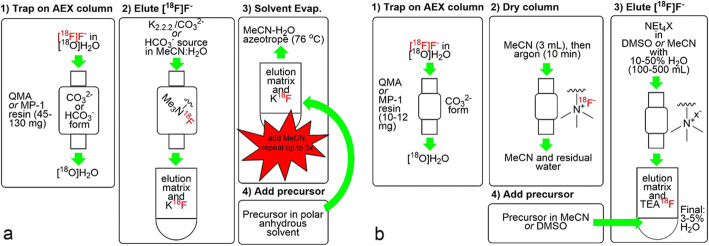
Traditional (**a**) and “non-anhydrous, minimally-basic” NAMB (**b**) approaches to the extraction, preparation and use of [^18^F]F^−^ for the manufacture of PET radiopharmaceuticals. X^−^ = HCO_3_^−^, ClO_4_^−^, or OTs^−^. This figure was obtained from Inkster et al. (2020) which was licensed under a Creative Commons License http://creativecommons.org/licenses/by/4.0/

The current ^18^F-labeling procedure uses several conservative steps: elution of [^18^F]fluoride from an anion-exchange column with a basic solution of K_2_CO_3_ or KHCO_3_ and Kryptofix®222 in a mixture of acetonitrile and water, followed by rigorous azeotropic drying (Fig. 1a). Although this technique is reliable, it still has some limitations: some precursor and ^18^F-labeled compounds are decomposed under basic conditions; variations in the drying step often influence the reproducibility of yields; and some [^18^F]F^−^ is lost due to volatilization and adsorption of the dried K[^18^F]F onto the walls of the reaction vessel during the drying process. To eliminate these limitations, a “non-anhydrous, minimally basic” (NAMB) approaches that simplifies the process and avoid the basic condition was proposed (Inkster et al. 2020) (Fig. 1b). In this system, [^18^F]F^−^ is efficiently eluted from a small amount of resin (10–12 mg) in an anion-exchange column with solutions of tetraethylammonium tosylate and perchlorate in polar aprotic solvents containing 10–50% water. Tosylate and perchlorate are minimally basic anions that do not contribute to the basicity of the reaction mixture. After dilution with an aprotic solvent, nucleophilic aromatic and aliphatic ^18^F-fluorination reactions were successfully accomplished. As shown by the demonstrative [^18^F]fallypride synthesis, this method can accommodate various volumes of aqueous [^18^F]F^−^ and concentrations of precursor, and is easily implemented with currently used automated synthesizers.

**Fig. 2:**
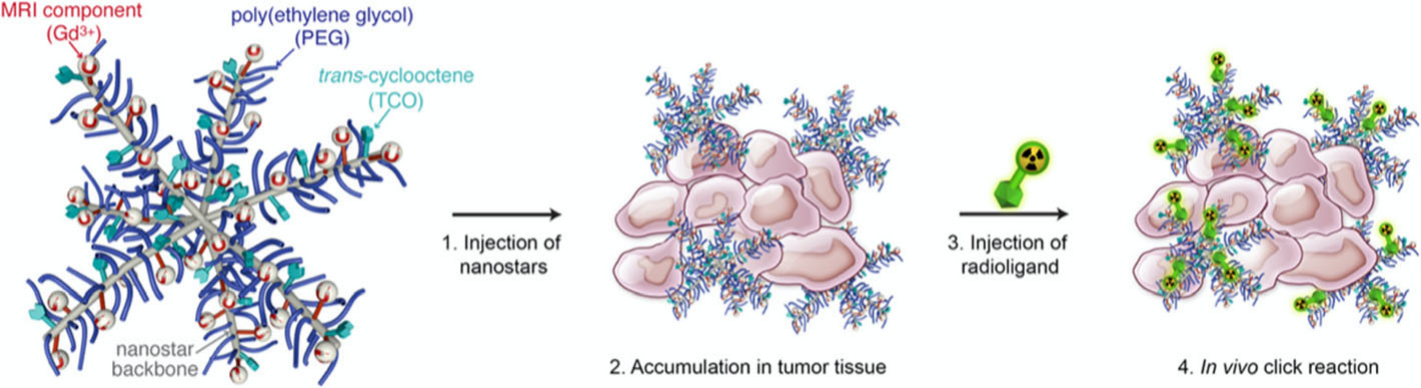
TCO-functionalized nanostars passively accumulate at the tumor site. After several days, a radioligand is injected that will bind to the TCO-functionalized nanostars at the tumor site via the bioorthogonal IEDDA click reaction. Unreacted radioligands are cleared from the blood circulation rapidly (within hours), resulting in minimal radiation doses to off-target tissues. (Reprinted from Goos et al. (2020), with permission from Elsevier)

**Development of pretargeting strategies for radiolabeled polymeric nanostars**

*By Ivan Penuelas*

Nanostars are star-shaped structures produced by crosslinking at one end of several linear polymeric arms. They can be easily synthetized and provide a high functionalization potential. The same group of authors has previously demonstrated the use of gadolinium (III)-tagged nanostars for MRI imaging and radiolabeling of nanostars both with iodine-125 and zirconium-89 using several elegant radiochemical approaches. In preclinical models, such nanostars highly accumulate in tumors via the enhanced permeability and retention effect.

In this paper, the authors move a step further, and apply a pretargeting strategy using the classical bioorthogonal cycloaddition click reaction between trans-cyclooctene (TCO)functionalized nanostars and fluorine-18 labelled tetrazine (Tz) as shown in Fig. 2 (Goos et al. 2020).

Comparing different tetrazines and adjusting the molecular distance between the TCOs and the polymer backbone, they tested this pretargeting approach in tumor bearing mice. Although tumor accumulation was suboptimal, the authors have developed a promising pretargeting strategy with nanostars that could allow for imaging and specific high-dose treatment of tumors using therapeutic radioisotopes.

**Visualizing AMPA receptors in vivo using [**^**11**^**C]K-2**

*By Jan Passchier*

**Fig. 3:**
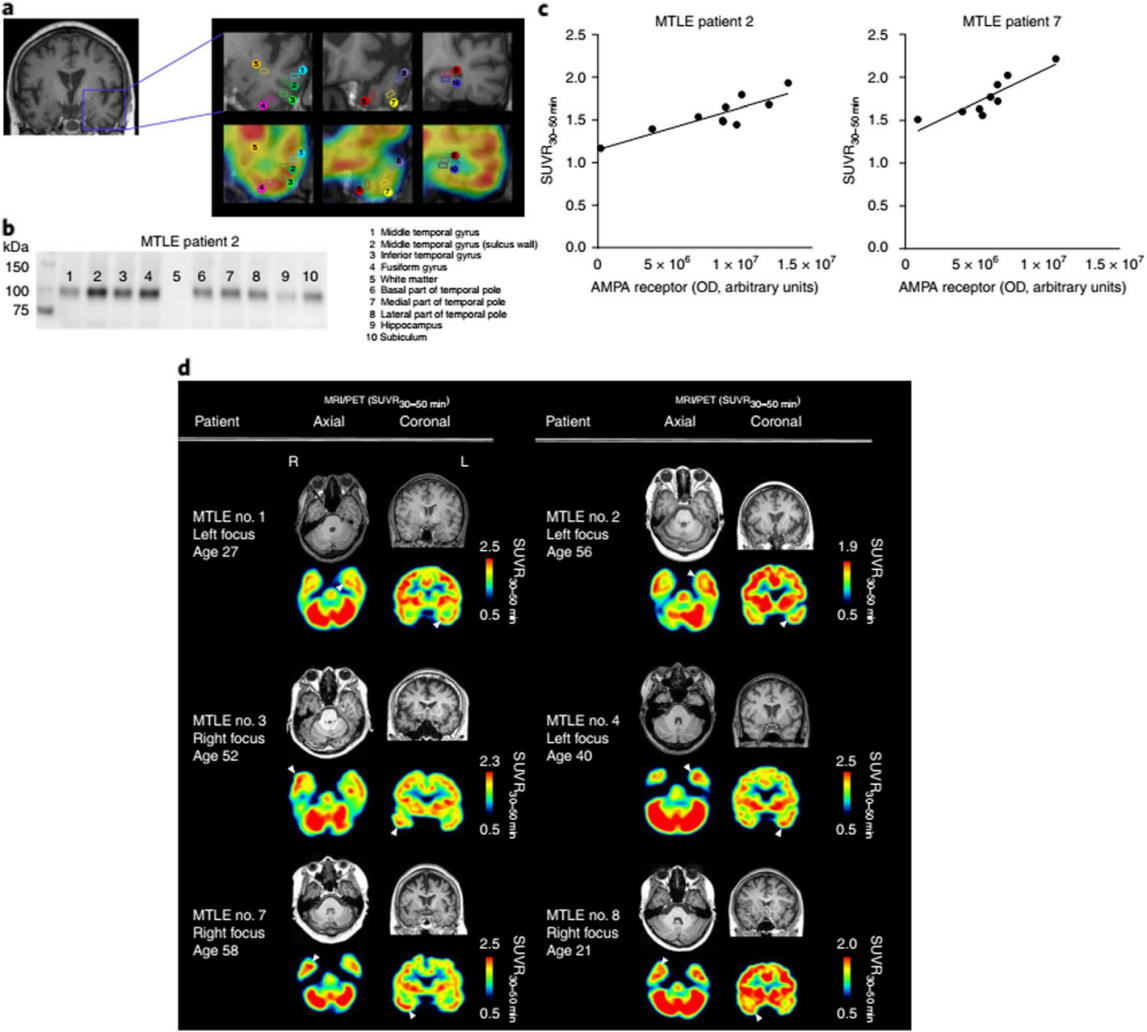
PET imaging characteristics of [^11^C]K-2 in patients with epilepsy. A: Presurgical MRI and PET images used to examine the correlation between AMPA receptor expression and retention of [^11^C]K-2 in patient with MTLE. VOIs on individual MRI images were defined within the outline of expected resection areas (left). B: Representative immunoblot of resected brain tissues in a patient with MTLE performed with a pan-AMPA receptor antibody. The origins of the samples are listed on the right, corresponding to the numbers on the blot and VOI identifications. C: Correlations between SUVR_30-50min_ for [^11^C]K-2 and the biochemical amount of AMPA receptor (OD) in multiple brain regions form patients with MTLE. D: SUVR_30-50min_ images of [^11^C]K-2 were obtained from 6 patients with MTLE. White arrows indicate the epileptogenic foci. This figure was obtained from Miyazaki et al. (2020) which was licensed under a Creative Commons License http://creativecommons.org/licenses/by/4.0/

^**99m**^**Tc/**^**188**^**Re analogy: a step forward in the way to a new theragnostic agent**

AMPA receptors are the main agents for fast excitatory synaptic transmission in the brain. It has long been known that dysregulation of AMPA receptors are associated with a range of neurological and psychiatric conditions. Considerable effort has been invested in identifying a suitable PET tracer to enable quantification of AMPA receptors in vivo but with little success. A recent paper by Miyazaki et al. looks to change this (Miyazaki et al. 2020). The authors found [^11^C]K-2, to demonstrate good specific binding in preclinical studies. Subsequent studies in healthy human subjects showed rapid radiotracer uptake in the brain and regional heterogeneity consistent with known AMPA receptor distribution. Based on Logan analysis, using white matter as reference region, the authors found BP_ND_ to range between 0.5–2 for grey matter regions. Strengthened by these results, an exploratory study in patients with mesial temporal lobe epilepsy (MTLE), scheduled for surgery, was conducted. This study found good correlation between increased [^11^C]K-2 uptake and AMPA receptor expression by Western blot (Fig. 3). The excellent work by the authors represents the first promising PET tracer to delineate AMPA receptors in vivo and has the potential to significantly further our understanding of disease and facilitate drug development programs.

*By Ana Rey*

Recently, a new concept based on targeting the tumour microenvironment and stroma instead of the tumour cells has been introduced for oncological imaging and therapy. Inhibitors of the fibroblast activating protein (FAPIs) labelled with ^68^Ga and ^177^Lu have demonstrated remarkable uptake in different tumour cell lines. However, they exhibited relatively short intratumoral half-lives, which limit their applicability for therapy. On the other hand, short-lived radionuclides like ^188^Re could deliver higher doses to the tumour. At the same time, the similarities between Tc and Re chemistry could be exploited to design potentially theragnostic tracers.

**Fig. 4:**
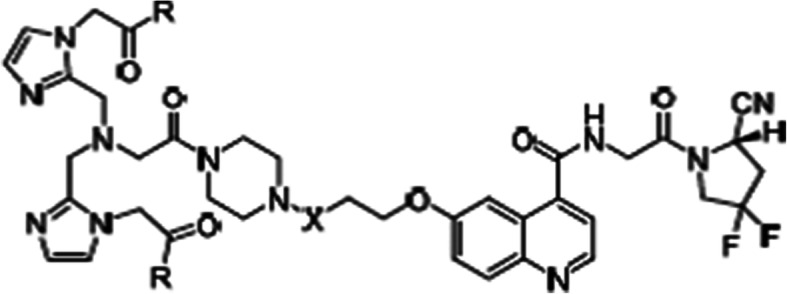
Structure of quinolone-based FAPIs for labelling with Tc/Re. This figure was originally published in JNM. Authors: Lindner et al. J Nucl Med. 2020 https://doi:10.2967/jnumed.119.239731

A family of quinolone-based FAPIs was presented (Lindner et al. 2020) attached to a polydentate chelator to coordinate the ^99m^Tc though the formation of Tc(I)tricarbonyl complexes (Fig. 4). The compounds have different linker lengths and modifications in the chelator through addition of hydrophilic amino acids to improve pharmacokinetics. The resulting ^99m^Tc-labelled FAPI tracers revealed excellent binding properties, high affinity and significant tumour uptake. A first-in-human study in patients with ovarian and pancreatic cancer showed promising results. Labelling experiments with ^188^Re are also planned to evaluate the potentiality as therapeutic radiopharmaceutical.

**Towards a simplified general** ^**11**^**C-labeling approach using a novel fluoride-mediated desilylation strategy**

*By Jean N. DaSilva*

**Fig. 5:**
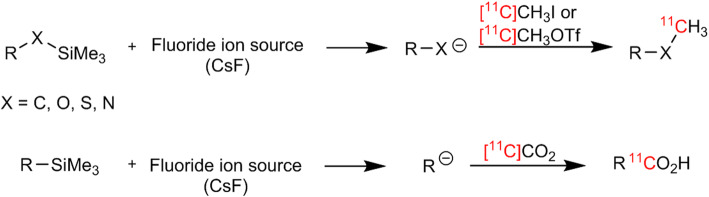
FMDS ^11^C-methylation and ^11^C-carboxylation*.* This figure was obtained from Qu et al. (2020) which was licensed under a Creative Commons License http://creativecommons.org/licenses/by/4.0/

Carbon 11 is a radionuclide of choice for probe synthesis as it can be incorporated into bioactive molecules without causing structural modification and biological property changes. Due to the short half-life, the introduction of C-11 needs to be done via a quick, efficient and robust method. Recently, the group from Weill Cornell Medicine reported a general novel fluoride-mediated desilylation (FMDS) approach to obtain ^11^C-labeled derivatives under mild conditions (Qu et al. 2020). This method involves generation of various nucleophiles in situ via desilylation of the organosilane analogs with fluoride anion sources (eg. CsF). The nucleophile can then quickly react with either [^11^C]methyl iodide/[^11^C]methyl triflate or [^11^C]CO_2_ to produce the corresponding ^11^C-labeled methylated or carboxylic acid derivative (Fig. 5). This paper reports ^11^C-labeling of various hybridized carbons, as well as oxygen, sulfur and nitrogen atoms with diversified functional groups. This simplified method provides several potential advantages as it requires no catalyst or base for the reaction. Furthermore, since most organosilanes and fluoride sources are not sensitive to atmospheric CO_2_, higher molar activities are expected for ^11^C-carboxylations. This FMDS-mediated procedure, although in its infancy, broadens the spectrum of radiochemical reactions available for ^11^C and is a promising strategy for the production of new ^11^C-labeled radiopharmaceuticals.

**Check [the] Points [out].**

*By Klaus Kopka*

This review article (Wierstra et al., 2019) highlights one of the current major questions in the field of radiopharmaceutical sciences within nuclear medicine: Can immune checkpoints, which have been targeted for years with biologicals e.g. with nivolumab or atezolizumab, be used as imaging biomarkers for non-invasive molecular imaging too? Within this context, the authors summarize the radiolabeled molecules employed so far pre-clinically and clinically for addressing a variety of checkpoints, e.g. PD-1, PD-L1, CTLA-4, and OX40. The authors discuss that with large molecules and their corresponding slow pharmacokinetics, radionuclides with a matching physical half-life are necessary.

In particular, the article discusses for which clinical questions during the course of a cancer disease, radiolabeled checkpoint ligands can be used for patient-stratification by means of non-invasive imaging. In addition to tissue biopsy, which leads to a decision on therapy, molecular imaging can non-invasively record the tumor heterogeneity [also over time], so that a biopsy can be planned in a more targeted manner. False-negative pathology could be minimized here. A therapy option involving the administration of checkpoint inhibitors could therefore be more personalized. Furthermore, the dynamic regulation of the immune checkpoints in the course of therapy [i.e. in my opinion over days, weeks, months, years] could be monitored optimally.

The future will show whether radiolabeled checkpoint ligandscan achieve clinical added value for the patients. Improvement of patient stratification by means of checkpoint PET/CT (and -SPECT/CT) appears obvious, especially in view of the increasing number of clinical trials and trials including combination therapies using already approved checkpoint inhibitors with concurrent additional treatment options.

Only if the clinical necessity is proven, the use of radiolabeled checkpoint ligands will be clinically established. So I can conclude: Check [the] points [on-tumor]!

^**11**^**C-Carbonylation chemistry may still have a bright future**

*By Sergio Todde*

The carbonyl group is widespread in organic molecules, being present in ketones, aldehydes, amides, esters, carboxylic acids, etc., and it may be labelled using [^11^C]CO as a radioactive precursor (Eriksson et al. 2020). [^11^C]CO is mainly obtained by passing cyclotron produced [^11^C]CO_2_ over zinc or molybdenum at 400° and 850 °C, respectively, with a 70–80% conversion rate. A major issue is [^11^C]CO transfer and concentration into the reaction vessel, that may be accomplished via high or low pressure methods, with the latter being easier to automate and to adapt to the GMP requirements. [^11^C]CO may then be used in a plethora of coupling reactions that lead to the preparation, often with suitable yield and molar activity, of many ^11^C-carbonylated compounds. However, only a small fraction of them have been tested in clinical trials, mainly due to both the lack of commercially available automated systems and the challenging methodology inherent to this interesting and potentially useful radiolabelling method.

**One-step** ^**18**^**F-fluorination applying enzyme-activatable and self-assembly strategy**

*By Mohammed Al-Qahtani*

Finding a suitable and reliable labeling method to infuse targeted tracer into a specific cells is an important aim for molecular imaging science. Taking advantages of the self-assembly of nanoscaled particles which could stimulate the accumulation of tracers in tumor cells, may serve as an efficient molecular imaging platform with enhanced and prolonged signal via enzyme-controlled condensation, and is a promising window to tackle this issue.

**Fig. 6:**
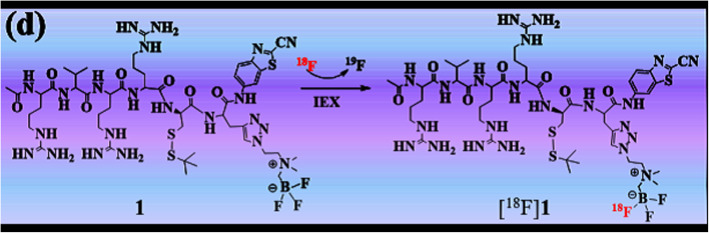
One-step labeling process of [^18^F]1. (Reprinted from Zhao et al. (2020), with permission from Elsevier)

**Fig. 7:**
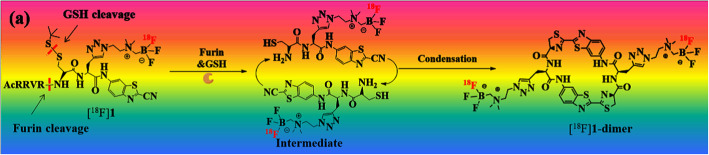
Condensation of [^18^F]1 after cleaved by furin and GSH. (Reprinted from Zhao et al. (2020), with permission from Elsevier)

In this article (Zhao et al. 2020), a furin-controlled self-assembly tracer [^18^F]**1** was designed and synthesized taking advantage of the biocompatible “click” reaction between cyanobenzothiazole analogues and cysteine. The functional group AMBF_3_ was introduced for one-step [^18^F]fluorination of the tracer (Fig. 6). The self-assembly mechanism of [^18^F]**1** could be attributed to the fact that the disulfide bond of Cys and the RVRR peptide motif in [^18^F]**1** can be cleaved by the intracellular GSH and furin, respectively. Then the condensation between the intermediates could happen spontaneously to yield a rigid and lipophilic dimer ([^18^F]**1**-dimer, Fig. 7), which would further self-assemble into nanoparticles ([^18^F]**1**-NPs) via π-π stacking interactions among dimers. The presented methodology is a new way of preparing a molecular imaging probes.

**An Alternative Approach for Imaging Neurodegeneration**

*By Robert Mach*

Most neurodegenerative disorders are characterized by the presence of insoluble protein aggregates that serve as a hallmark of disease. Alzheimer’s disease (AD) is characterized by the formation of two insoluble protein aggregates, amyloid beta (Abeta) plaques and tau-enriched neurofibrillary tangles (NFTs). The synucleinopathies (i.e., Parkinson’s disease (PD) and multiple system atrophy (MSA) are characterized by the formation of aggregated alpha synuclein in the form of Lewy bodies, Lewy neurites, and glial cell inclusions. The development of PET probes for Abeta plaques and tau-based NFTs has achieved tremendous success, as opposed to alpha synuclein, which still represents an unmet need. An alternative strategy for imaging neurodegeneration is the use of PET probes that can measure synaptic density via SV2A, an essential vesicle membrane protein expressed in all presynaptic terminals. In this paper (Matuskey et al. 2020), the investigators compared the uptake of the SV2A radiotracer [^11^C]UCB-J in PD patients and age-matched controls. A significant reduction was observed in the substantia nigra and other brain structures that are affected in PD. A key observation was the reduced binding of the radiotracer in locus coerulus and dorsal raphe, regions containing norepinephrine and serotonin neurons, demonstrating that the pathology of PD extends beyond the nigrostriatal dopaminergic system. The PET results were consistent with autoradiography studies conducted with [^3^H]UCB-J in postmortem samples from a separate cohort of PD and age-matched controls. The results of this study were also similar to earlier studies in AD patients reported by this group (Toyonaga et al. 2019). The methods described in this paper provide a novel strategy for imaging neurodegenerative disorders with PET, one that is more congruent with the loss of synaptic function that underlies the cognitive and motor deficits found in AD and PD.

**New prosthetic group key to effective orthogonal** ^**18**^**F-radiolabeling of peptides and proteins**

*By Peter J.H. Scott*

**Fig. 8:**
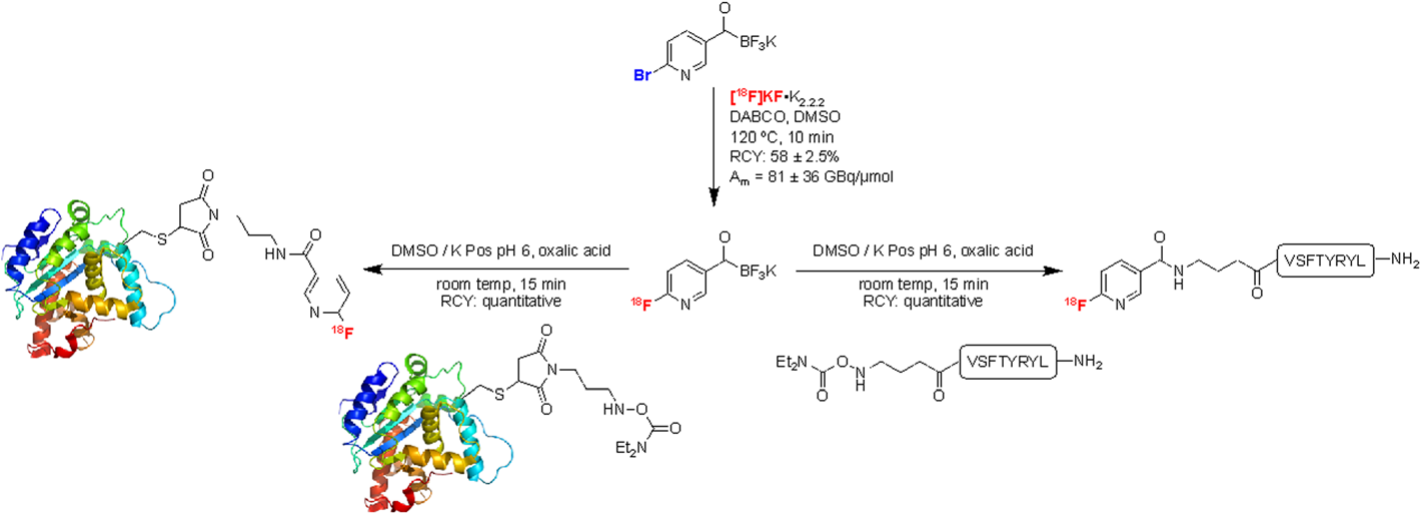
Radiolabeling of peptides and proteins using 6-[^18^F]fluoropyridyl acyltrifluoroborate ([^18^F]FPAT). This figure was originally published by Chiotellis et al. (2020) this article was licensed under a Creative Commons Attribution-NonCommercial 3.0 Unported License and published by The Royal Society of Chemistry

The introduction of immunotherapy for cancer has necessitated development of tools for immunoimaging (Mayer and Gambhir 2018). These tools are often labeled biologics (e.g. antibodies, peptides, proteins) and new strategies to radiofluorinate them are emerging (Specklin et al. 2019). In the case of peptides and proteins it is typical to radiolabel them prior to injection with ^18^F, directly or via a prosthetic group, but such labeling can be difficult because of harsh reaction conditions. To overcome this, Chiotellis and colleagues report chemoselective ^18^F-incorporation into pyridyl acyltrifluoroborates, and demonstrate rapid (15 min) and quantitative radiolabeling of peptides and proteins through an amide bond (via hydroxylamine precursors) at room temperature under mild aqueous conditions as shown in Fig. 8 (Chiotellis et al. 2020). Common protein and peptide functionality is tolerated without need for protecting groups, suggesting the orthogonal radiolabeling strategy could become a method of choice for radiofluorinating peptides and proteins in the future.

**Cool (room temperature) fluorine-18 substitute and release strategy for peptide labeling**

*By Guy Bormans*

There is still a high need for novel methods to fully open up the fluorine-18 radiochemical space towards (thermo labile) peptides and proteins. Several approaches are already available including prosthetic group approaches (e.g N-succinimidyl-4-[^18^F]fluorobenzoate ([^18^F]SFB) or fluorine-18 labeling via silicon, boron or aluminium-[^18^F]fluoride bond formation. Radiofluorination using aromatic nucleophilic substitution on the pyridine ring using trimethylammonium as leaving group has already been applied successfully, but the synthesis of the corresponding trimethylammonium precursors can be problematic. Mylène Richard et al. from the BioMaps CEA group in Paris reported a novel approach for the facile synthesis of quaternary ammonium precursors by reaction of tertiary amines with 2-triflyl pyridines (Richard et al. 2020). This approach allowed high yield synthesis of triflate salts of “DABCO-nium” pyridine-derivatised peptides that were labeled with high radiochemical yields (40–50%) at 40 °C in DMSO.

**Fig. 9:**
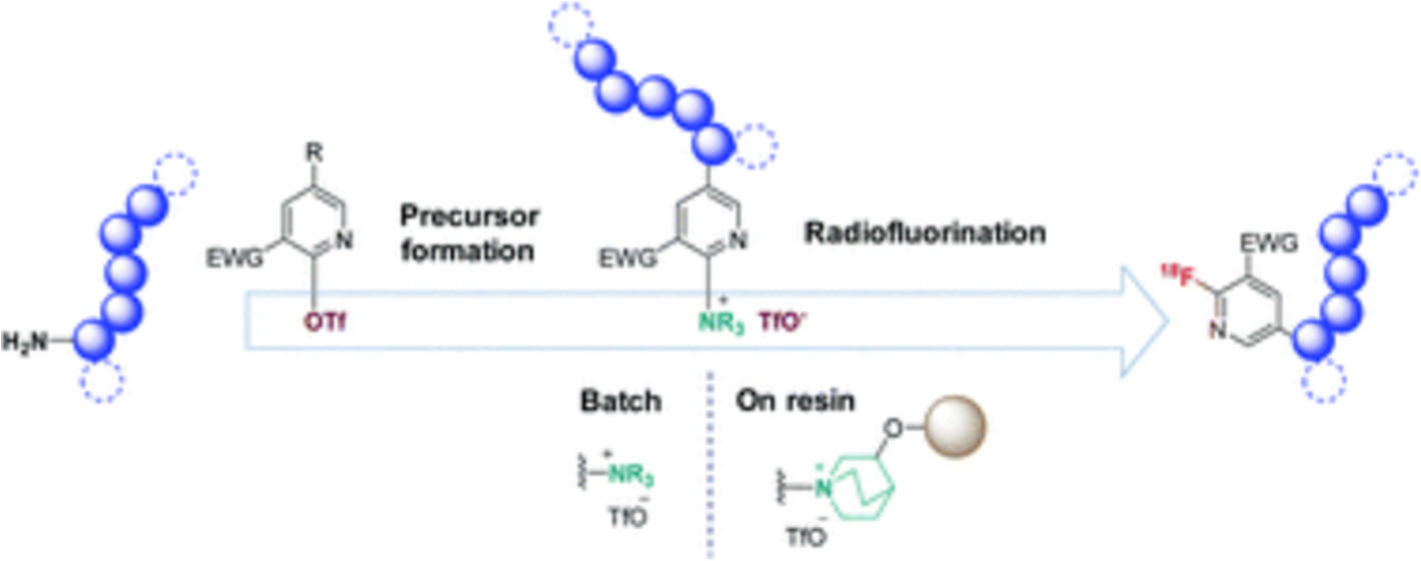
Resin-based ^18^F-radiofluorination for synthesis of [^18^F]fluoropyridine. This figure was originally published by Richard et al. (2020) this article was licensed under a Creative Commons Attribution-NonCommercial 3.0 Unported License and published by The Royal Society of Chemistry

The methodology was taken a step further by reaction of an (aminomethyl)polystyrene resin bound quinuclidine with triflyl pyridine yielding the corresponding resin bound alkylammonium pyridine. The modified resin was used to trap [^18^F]fluoride that substitutes the alkylammonium group, releasing the corresponding [^18^F]fluoropyridine from the resin. Although radiochemical yields should further be improved, this approach provides a fast, simple, clean and elegant method for labeling of various compounds including peptides with fluorine-18 (Fig. 9).

**Easy transfer of an** ^**18**^**F-somatostatin receptor ligand into GMP compliant routine application**

*By Marianne Patt*

The development of ^18^F-labelled tracers of neuroendocrine tumors for diagnostic care is of high relevance for current clinical nuclear medicine. Why? Restriction to ^68^Ga-labelled ligands limits cost-effective production and therefore availability. The translation into clinical routine has severe implications on the comprehensiveness of validation activities in order to comply with current GMP requirements. This article (Tshibangu et al. 2020) describes state of the art methods and provides valuable results of the validation of analytical methods for an ^18^F-labelled analogue of the somatostatin receptor ligand NOTA-TOC, [^18^F]AlF-NOTA-octreotide (Laverman et al. 2010, 2012). In addition, a robust production method on a commercially available synthesis platform is described, as well as preclinical data on metabolite studies obtained with the tracer are provided

**First achievements towards direct** ^**18**^**F-trifluoromethylation of native amino acids in unmodified small proteins**

*By Danielle Vugts*

PET imaging of fast clearing biologicals such as nanobodies, domain antibodies, diabodies is preferably done with short-lived radionuclides such as fluorine-18. This can for example be achieved by reacting the fluorine-18 labelled prosthetic group [^18^F]SFB with a lysine molecule in the biological, or via a two-step strategy, in which first a reactive group such as a DBCO is coupled to the biological, followed by reaction with a fluorine-18 labelled tetrazine. The first method suffers from poor conjugation yields, and the second requires premodification of the biological. Ideally, fluorine-18 is introduced directly without premodification, with high high yields at ambient temperature. A method was developed to introduce a [^18^F]trifluoromethyl group using Langlois’ reagent onto the native aromatic peptide residues tyrosine and tryptophan (Kee et al. 2020). The method was nicely applied to multiple amino acids, peptides (including somatostatin-14) and recombinant insulin. Although applicable to the example protein and peptides, the drawback of the method is the relatively high Fe and DMSO concentration needed for good radiochemical yields. Further improvements to make this a general fluorine-18 labeling strategy are therefore highly desired.

## Conclusions

Trends in radiochemistry and radiopharmacy are highlighted demonstrating the progress in the research field being the scope of EJNMMI Radiopharmacy and Chemistry.

## Data Availability

Datasets mentioned in this article can be found in the cited articles.

## Abbreviations

AD: Alzheimer’s Disease
AMPA: α-amino-3-hydroxy-5-methyl-4-isoxazolepropionic acid
DBCO: Dibenzo cyclo-octyne
CT: Computed Tomography
FAPI: Fibroblast activation protein inhibitors
FET: O-(2-[^18^F]fluoroethyl)-L-tyrosine
FMDS: Fluoride mediated desilylation
GMP: Good Manufacturing Practice
GSH: Glutathion
IEDDA: Inverse Electron Deficient Diels Alder
MAS: S-acetylmercaptoacetyltriserine
MRI: Magnetic Resonance Imaging
MSA: Multiple System Atrophy
NFT: Neurofibrillary Tangles
NIR: Near Infrared
PARP: Poly-ADP-Ribose-Polymerase
PD: Parkinson’s Disease
PEG: Poly Ethylene Glycol
PET: Positron Emission Tomography
PSMA: Prostate Specific Membrane Antigen
SFB: S-Fluorobenzoyl succinimide
SPECT: Single Photon Emission Computed Tomography
TCO: Trans Cyclo Octene
Tz: Tetrazine
